# Treatment of neonatal septic arthritis sequelae of hip: a case report

**DOI:** 10.4076/1757-1626-2-6332

**Published:** 2009-07-01

**Authors:** Cen Bytyçi, Hasime Qorraj, Dafina Bytyqi

**Affiliations:** 1 Orthopaedic Department, University Clinical CenterPrishtinaKosovo; 2 Faculty of Medicine, University of PrishtinaPrishtinaKosovo

## Abstract

**Introduction:**

The most serious complication of the septic arthritis of the hip in childhood and especially in newborns is the avascular necrosis of the femoral head. The aim of the study was evaluation of residual deformity after neonatal septic arthritis of the hip in a boy aged thirteen years.

**Case presentation:**

A 13-year-old, white male, was operatively treated by intertrochanteric osteotomy of valgisation, anterotation and extension at age of twelve years because of leg length discrepancy, changes in the femoral neck, coxa vara, plana and breva. It was delay in diagnosis and failure to begin treatment promptly in the neonatal period.

**Conclusion:**

Valgus intertrochanteric osteotomy of the femur, moves the greater trochanter distally and laterally, tensioning the abductors muscles and improving their leverage.

## Introduction

The most serious complication of the septic arthritis of the hip in childhood and especially in newborns is the avascular necrosis of the femoral head, which can lead to partial or complete destruction of the capital femoral epiphysis, the growth plate or both [1]. The aim of the treatment of sequelae of neonatal septic arthritis of the hip is to preserve good relation between the femoral head and acetabulum.

## Case presentation

**Figure 1.**
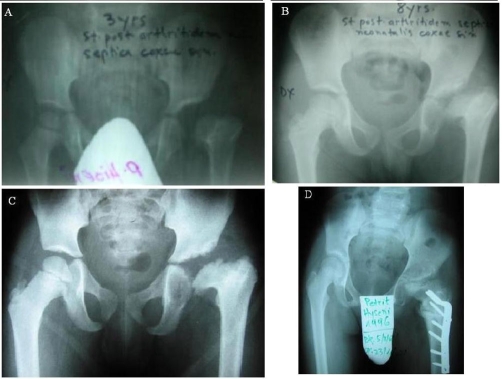
Late metaphyseal changes in the femoral neck, coxa vara, plana and breve associated with septic arthritis of the hip. **(A)** Three years after the onset of septic arthritis on the left hip. **(B)** Sequelae of acute septic arthritis of the hip at the age of eight. **(C)** Ten-year-old, the result before an abduction intertrochanteric osteotomy. **(D)** Twelve years old, the result after an abduction intertrochanteric osteotomy.

We present a case of a 13-year-old boy, complaining of painless limp on the left hip. He had suffered septic arthritis of the left hip in the neonatal period. At follow-up from 3 to 13 years, a trend towards severe deformities was seen (Figure 1 A, B). On clinical examination, there was muscle wasting at the left hip and thigh region. The patient had Duchenne-Trendelenburg limp and the Trendelenburg sign with flexion, rotational and adduction contracture of the left hip. Abduction and adduction was tested with patient supine.

The Thomas test was positive and flexion deformity was 25°, abduction 20°, adduction deformity of 25°. Internal and external rotation was determined with the patient prone. Internal rotation was limited of 20°. Determination of leg length discrepancy was done with patient laying and standing, and the shortening was 3.8 cm. Magnetic Resonance Imaging (MRI) was not done because our hospital lacks the equipments. According to the system suggested by Choi at al [2] our patient was grouped under Choi’s Type IIIA, (Figure 1C) with severe coxa vara angular deformity with retroversion but no pseudoarthrosis of the femoral neck. Intertrochanteric osteotomy was done at the age of twelve with lateral approach to change the loading of the hip and to place the epiphyseal plate at right angle to the resultant of the compressive forces. With this intertrochanteric osteotomy of valgisation of 35° with anterotation of 10° and extension 25° we achieved transferring the greater trochanter distally and laterally so it is level with the center of the femoral head, restoring normal tension to the pelvitrochanteric muscles and improving their mechanical efficiency (Fig.1 D). With this procedure, we placed the superior end of the femur against the lateral aspect of the pelvis and also increased the distance between the tip of the trochanter and the center of the hip rotation.

## Conclusion

Delay in diagnosis, failure to begin treatment promptly, and patient age less than 1 year are the most common reason for late complication [3-6]. Acute septic arthritis represent surgical emergency, which demands early and vigorous treatment in order to preserve normal joint function [7,8]. In our case, intertrochanteric femoral osteotomy increased the stability of the hip due to correction of the neck-shaft angle. In this type of osteotomy the operation is extracapsular so the hip joint is not directly approached. The intertrochanteric osteotomy in our case has given a satisfactory result, improving the lower-extremity length discrepancy from 3.8 cm preoperatively to 1.2 cm postoperatively. The pelvic drop (Trendelenburg gait) was also reduced.

## Abbreviations

MRI: Magnetic Resonance Imaging
